# A Machine Learning-Based Prediction of Hospital Mortality in Patients With Postoperative Sepsis

**DOI:** 10.3389/fmed.2020.00445

**Published:** 2020-08-11

**Authors:** Ren-qi Yao, Xin Jin, Guo-wei Wang, Yue Yu, Guo-sheng Wu, Yi-bing Zhu, Lin Li, Yu-xuan Li, Peng-yue Zhao, Sheng-yu Zhu, Zhao-fan Xia, Chao Ren, Yong-ming Yao

**Affiliations:** ^1^Trauma Research Center, Fourth Medical Center of the Chinese PLA General Hospital, Beijing, China; ^2^Department of Burn Surgery, Changhai Hospital, Naval Medical University, Shanghai, China; ^3^School of Mathematics and Statistics, Beijing Institute of Technology, Beijing, China; ^4^School of Computer Science and Technology, Wuhan University of Technology, Wuhan, China; ^5^Department of Cardiothoracic Surgery, Changzheng Hospital, Naval Medical University, Shanghai, China; ^6^Medical Research and Biometrics Center, Fuwai Hospital, National Center for Cardiovascular Diseases, Chinese Academy of Medical Sciences and Peking Union Medical College, Beijing, China; ^7^Department of General Surgery, First Medical Center of Chinese PLA General Hospital, Beijing, China

**Keywords:** postoperative sepsis, intensive care unit, extreme gradient boosting, coagulation, prediction

## Abstract

**Introduction:** The incidence of postoperative sepsis is continually increased, while few studies have specifically focused on the risk factors and clinical outcomes associated with the development of sepsis after surgical procedures. The present study aimed to develop a mathematical model for predicting the in-hospital mortality among patients with postoperative sepsis.

**Materials and Methods:** Surgical patients in Medical Information Mart for Intensive Care (MIMIC-III) database who simultaneously fulfilled Sepsis 3.0 and Agency for Healthcare Research and Quality (AHRQ) criteria at ICU admission were incorporated. We employed both extreme gradient boosting (XGBoost) and stepwise logistic regression model to predict the in-hospital mortality among patients with postoperative sepsis. Consequently, the model performance was assessed from the angles of discrimination and calibration.

**Results:** We included 3,713 patients who fulfilled our inclusion criteria, in which 397 (10.7%) patients died during hospitalization, and 3,316 (89.3%) patients survived through discharge. Fluid-electrolyte disturbance, coagulopathy, renal replacement therapy (RRT), urine output, and cardiovascular surgery were important features related to the in-hospital mortality. The XGBoost model had a better performance in both discriminatory ability (c-statistics, 0.835 vs. 0.737 and 0.621, respectively; AUPRC, 0.418 vs. 0.280 and 0.237, respectively) and goodness of fit (visualized by calibration curve) compared to the stepwise logistic regression model and baseline model.

**Conclusion:** XGBoost model has a better performance in predicting hospital mortality among patients with postoperative sepsis in comparison to the stepwise logistic regression model. Machine learning-based algorithm might have significant application in the development of early warning system for septic patients following major operations.

## Introduction

Sepsis is a severe complication following major surgery and responsible for poor outcomes of postoperative patients by inducing multiple organ dysfunction and increasing in-hospital mortality. Although great progress has been made in the early recognition and therapeutic strategies, the incidence and mortality of septic complications remain unacceptably high (1–3). It has been documented that there are ~30% of septic patients after surgical procedures, and the number of patients who developed postoperative sepsis increases annually (4–6). Given the high incidence and poor prognosis, the Agency for Healthcare Research and Quality (AHRQ) defined the “postoperative sepsis” as a critical indicator for patients' safety, which mainly focused on preventable surgical complications and iatrogenic events after surgical procedures (7, 8).

Various evidences have demonstrated that immunocompromised state is strongly associated with the pathogenesis of postoperative sepsis (9). For example, impaired antigen presenting capacity of monocytes and dominant differentiation of type 2 helper T cells were all characterized in the animal models of postoperative sepsis (10–12). Meanwhile, researchers identified disparate gene expression profiles of whole blood cells from surgical patients with or without postoperative sepsis, and found that the expression patterns of interleukin (IL) 1β (IL-1β), tumor necrosis factor (TNF) superfamily, member 2, and CD3D were significantly different (13). However, the “Surviving Sepsis Campaign” (SSC) guidelines didn't provide distinctive treatments for postoperative sepsis (14). Moreover, there were insufficient clinical trials that specifically testified the guidelines in the postoperative sepsis cohort. Most of the studies examined the short-term mortality in septic patients admitted to emergency department or intensive care unit (ICU) that covered multiple types of sepsis (8, 15, 16). On the contrary, few studies specifically characterized the clinical outcomes of patients with postoperative sepsis.

In the present study, we aimed to establish a predictive model on in-hospital mortality among patients with postoperative sepsis. Given the limitation of conventional statistical methods in processing retrospective data that contained covariates of high correlation and inevitable missing values, we enrolled advanced machine learning algorithm, called extreme gradient boosting (XGBoost), to identify the important clinical features for predicting in-hospital mortality.

## Materials and Methods

### Database

Medical Information Mart for Intensive Care-III (MIMIC-III), a large online critical care database, was applied for the current study (17). Of note, MIMIC-III was a comprehensive dataset which contained clinical data of all the patients admitted to ICU of Beth Israel Deaconess Medical Center (BIDMC) in Boston, Massachusetts, from 2001 to 2012. In brief, it included more than fifty thousand distinct adult (aged >16 years) ICU patients and approximately eight thousand neonate cases. We had obtained the permission for accessing the database after the completion of “Protecting Human Research Participants,” an online training course launched by National Institutes of Health (NIH) (certification number: 32450965). We conducted this study in accordance with the Transparent Reporting of a multivariable prediction model for Individual Prognosis or Diagnosis (TRIPOD) recommendation (18).

### Study Population

The selection of patients was based on “postoperative sepsis” criteria proposed by AHRQ combining with Sepsis 3.0 criteria, in which sepsis was diagnosed by sequential organ failure assessment (SOFA) score ≥2 plus documented or suspected infection (7, 19). Additionally, infection was confirmed in accordance with ICD-9 code in the MIMIC-III database. In this study, we included all patients (aged >18 years) who underwent surgical procedures prior to ICU admission and fulfilled Sepsis 3.0 criteria within 24 h post ICU admission. Patients were excluded even if they were in line with AHRQ selection criteria: (1) who had a principal or secondary diagnosis of sepsis or infection on admission; (2) who were diagnosed with cancer and had other immunocompromised state, including hematologic malignancies, HIV, prolonged usage of corticosteroids, and organ transplantation; (3) who were admitted to ICU with pregnancy, childbirth, or puerperium; (4) who stayed in hospital <4 days; (5) who had incomplete or unobtainable medical data records on admission.

### Variables Extraction and Outcome Measurement

Clinical and laboratory variables were collected within the first 24 h after ICU admission. Demographic data was obtained, including age, gender, body mass index (BMI), and elective surgical type. Laboratory findings, including white blood cell (WBC) counts, hematocrit, platelet counts, glucose, lactate, creatinine, blood urea nitrogen (BUN), coagulation profile, chloride, potassium, sodium, bicarbonate, albumin, bilirubin, partial pressure of arterial oxygen (PaO_2_), partial pressure of arterial carbon dioxide (PaCO_2_), total CO_2_, and pH were incorporated. In addition, vital signs, including blood pressure, respiratory rates, heart rates, and body temperature were included. Comorbidities, such as congestive heart failure, cardiac arrhythmia, neurological disorders, diabetes, anemia, and obesity, were also recorded. Prognostic scoring systems, including SOFA score, Oxford Acute Severity of Illness Score (OASIS), Simplified Acute Physiology Score II (SAPSII), and Glasgow Coma Scale (GCS) were calculated and analyzed by using variables obtained in the first 24 h during admission. Notably, both the maximum and minimum values of some indicators were collected and analyzed for multiple measurements.

As severe data missing might render bias, all eligible predictors were screened, and variables with more than 30% missing values were not taken into subsequent model establishment. Correspondingly, we conducted multivariate imputation for variables with <30% missing values.

We chose in-hospital mortality as our primary endpoint, which was defined as survival status at hospital discharge. Patients without outcome information were excluded from the final cohort.

### Statistical Analysis

Baseline characteristics of enrolled participants were presented and compared between survivors and non-survivors by applying either Student *t*-test, Chi-square test and Mann-Whitney *U*-test as appropriate. Continuous variables were characterized as mean (standardized differences [SD]) or median (interquartile range [IQR]), while categorical or ranked data were reported as count and proportion.

We employed stepwise logistic regression model to select predictors of in-hospital mortality. Both forward and backward directions were used in variable selection processes, in which Akaike Information Criterion (AIC) was applied as the selection criteria of the optimal model.

Furthermore, we applied Extreme Gradient Boosting (XGBoost) model to predict in-hospital death among patients with postoperative sepsis. XGBoost was a machine learning algorithm, which mainly functioned as iterative refit of weak classifier to residuals of previous models, meaning that the current weak classifier was generated based on previous one in order to optimize the predictive efficiency (20, 21). In each round of iteration, it focused more on misclassified observations. As eligible variables were included into the model, it outputted the importance score of each variable. Meanwhile, XGBoost could automatically process missing data through assigning a default direction to the null values. To reach the optimal model performance of XGBoost, we assessed and tuned the hyperparameters, including learning rates, maximum depth of a tree, number of estimators, alpha, and lambda. In this study, the original dataset was randomly divided into 5 subsets. One-fold was used as testing subset, while the other four-fold were processed to tune the hyperparameters, in which 25% were applied for calibration, and four-fold cross validation with grid search was conducted in remaining 75% of data. The hyperparameters with the highest area under the receiver operator characteristic curve (AUROC) were selected. The sufficiently tuned XGBoost hyperparameters were subsequently added back for training and calibrating the model, which was further validated in one held-out testing subset (22). Detailed process for tuning hyperparameters was provided in Supplemental Figure S1.

Model performance of both models was assessed in multiple dimensions. To test discriminatory ability, we used receiver operating characteristic (ROC) curve and c-statistic. Meanwhile, calibration plot revealed the correlation between observed and predicted risk, which was applied to evaluate the goodness of fit. The area under the precision-recall curve (AUPRC) provides a robust metric for unbalanced datasets, which has been a critical measure in assessing model performance. Given that, precision-recall curve with AUPRC, accuracy and recall were also applied to evaluate the performance of models. Of note, SOFA score that was commonly used for evaluating the severity of septic patients was assigned as a baseline model, and compared with stepwise logistic regression and XGBoost models as well. Aforementioned statistical analyses were performed by using IBM SPSS Statistics software (version 23.0), Python software (version 3.4.3), and R software (version 3.6.1). Two tailed *P* < 0.05 was deemed as statistical significance.

## Results

### Participants

**Figure 1:**
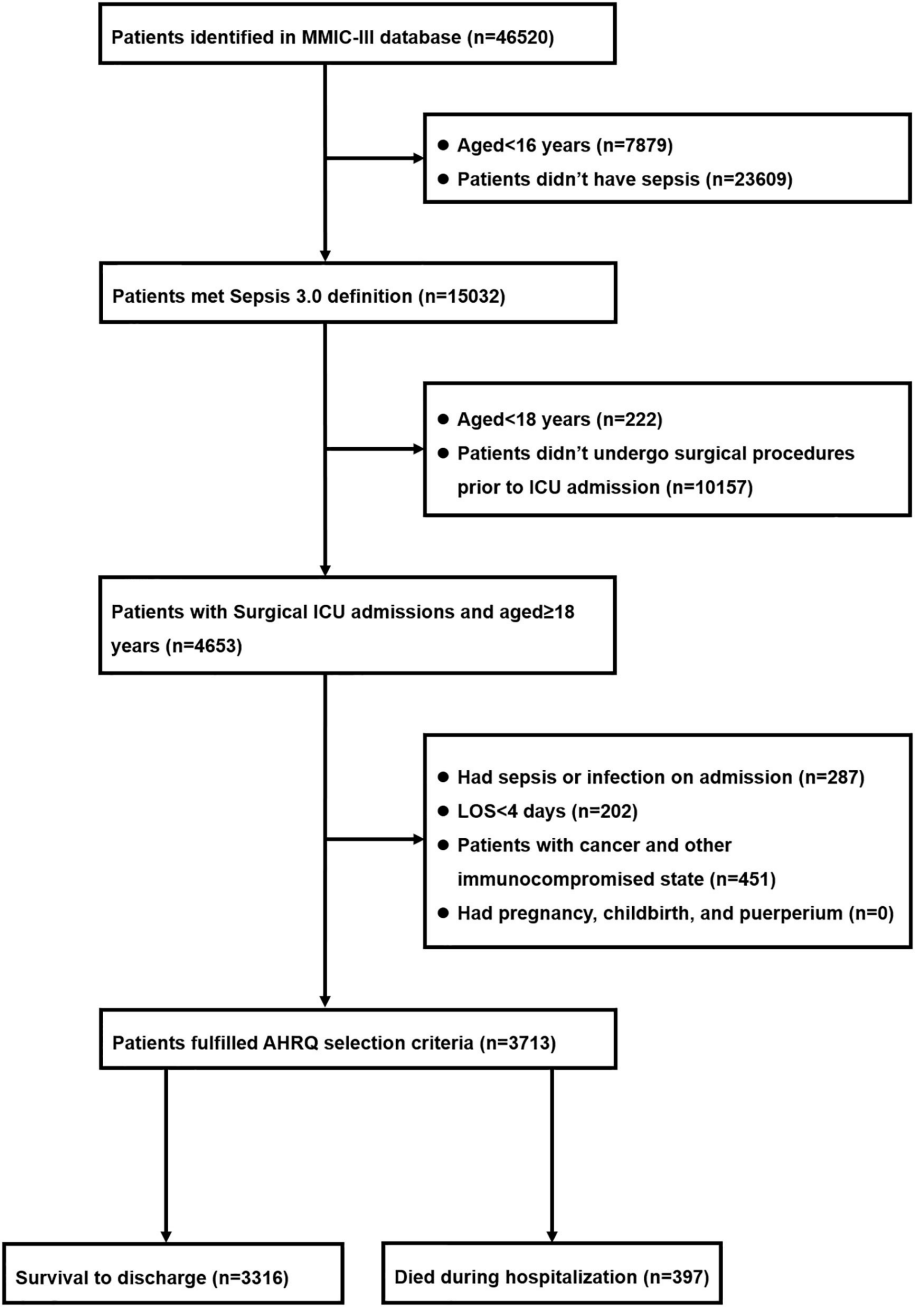
Flow diagram of patient inclusion.

Among 46,520 patients in the MIMIC-III database, 15,302 of them met with Sepsis 3.0 criteria. There were 4,653 potentially eligible adult patients (aged ≥18 years) who underwent surgical procedures prior to ICU admission. After excluded 940 patients in accordance with the AHRQ exclusion criteria, 3,713 patients were deemed to develop postoperative sepsis and were eventually incorporated into the study cohort, in which 397 (10.7%) patients died during hospitalization and 3,316 (89.3%) of them survived through discharge. The detailed information with regard to the enrollment and selection process was presented in Figure 1.

The comparison of baseline characteristics between survivors and non-survivors was summarized in Table 1. Notably, patients of the non-survivor group were much older than those of the survivor group (86.6 ± 59.2 vs. 74.6 ± 48.0; *P* = 0.001). As for the comorbidities, patients with postoperative sepsis who died during hospitalization had higher incidence of congestive heart failure (40.6 vs. 31.2%; *P* < 0.001), cardiac arrhythmias (48.1 vs. 39.4%; *P* = 0.001), renal failure (24.7 vs. 15.8%; *P* < 0.001), coagulopathy (30.5 vs. 13.0%; *P* < 0.001), and digestive disorders (16.9 vs. 7.8%; *P* < 0.001). The maximum respiratory rates (29.0 ± 8.1 vs. 27.7 ± 6.7; *P* = 0.002) were significantly higher in patients from the non-survivor group, while the minimum systolic blood pressure (BP) (84.1 ± 18.3 vs. 89.0 ± 16.2; *P* < 0.001), minimum diastolic BP (55.6 ± 9.7 vs. 58.3 ± 9.4; *P* < 0.001), and minimum mean BP (52.7 ± 14.1 vs. 56.5 ± 12.5; *P* < 0.001) were lower than those from the survivor group. Compared to survivors, non-survivors had higher levels in blood lactate (2.9 [IQR: 1.8, 5.8] vs. 2.3 [IQR: 1.5, 3.8]; *P* < 0.001), BUN (30 [IQR: 20, 46] vs. 21 [IQR: 15, 32]; *P* < 0.001), and creatinine (1.4 [IQR: 0.9, 2.4] vs. 1.1 [IQR: 0.8, 1.6]; *P* < 0.001). Additionally, higher international normalized ratio (INR) (1.9 ± 1.8 vs. 1.6 ± 1.1; *P* = 0.001), longer prothrombin time (PT) (16.1 [IQR: 14.1, 20.0] vs. 15.2 [IQR: 13.7, 17.3]; *P* < 0.001), and activated partial thromboplastin time (APTT) (39.5 [IQR: 30.6, 71.0] vs. 35.2 [IQR: 29, 48.2]; *P* < 0.001) were noted among patients in the non-survivor group when compared to those in the survivor group.

**Table 1:** Baseline characteristics between survivors and non-survivors.

**Characteristics**	**Survivors**	**Non-survivors**	***P*-value**
	**(*n* = 3,316)**	**(*n* = 397)**	
**Demographic characteristics**			
Age, mean (SD)	74.6 (48.0)	86.6 (59.2)	<0.001
Gender female, *n* (%)	1,511 (45.6)	170 (42.8)	0.299
BMI, mean (SD)	29 (7.6)	28.1 (7.4)	0.055
**Elective surgical type**, ***n*** **(%)**			0.029
Cardiovascular surgery	1,421 (42.9)	140 (35.3)	
Neurosurgery	462 (13.9)	67 (16.9)	
Orthopedic surgery	178 (5.4)	16 (4.0)	
Thoracic surgery	155 (4.7)	19 (4.8)	
Plastic surgery	23 (0.7)	2 (0.5)	
Others	1,077 (32.5)	153 (38.5)	
**Comorbidities**, ***n*** **(%)**			
Congestive heart failure	1,034 (31.2)	161 (40.6)	<0.001
Cardiac arrhythmias	1,306 (39.4)	191 (48.1)	0.001
Diabetes	1,018 (30.7)	112 (28.2)	0.309
Renal failure	525 (15.8)	98 (24.7)	<0.001
Coagulopathy	430 (13.0)	121 (30.5)	<0.001
Digestive disorders	258 (7.8)	67 (16.9)	<0.001
Mechanical ventilation, *n* (%)	2,259 (68.1)	284 (71.5)	0.167
Renal replacement therapy, *n* (%)	153 (4.6)	49 (12.3)	<0.001
**Prognostic scoring system, median (IQR)**			
SOFA	5 (3, 7)	6 (4, 10)	<0.001
SAPS II	37 (30, 45)	47 (40, 57)	<0.001
OASIS	33 (27, 39)	39 (33, 44)	<0.001
**Vital signs, mean (SD)**			
Maximum heart rates (/min)	105.4 (19.4)	107.6 (23.4)	0.074
Minimum systolic BP (mmHg)	89.0 (16.2)	84.1 (18.3)	<0.001
Minimum diastolic BP (mmHg)	58.3 (9.4)	55.6 (9.7)	<0.001
Minimum mean BP (mmHg)	56.5 (12.5)	52.7 (14.1)	<0.001
Maximum respiratory rates (/min)	27.7 (6.7)	29.0 (8.1)	0.002
Maximum temperature (°C)	37.7 (0.8)	37.6 (0.8)	0.024
**Laboratory findings**			
Minimum WBC (×10^9^/L, median [IQR])	10.2 (7.2, 13.9)	11.1 (7.8, 15.6)	0.005
Minimum platelet (×10^9^/L, median [IQR])	177 (119, 253)	149 (89, 234)	<0.001
Maximum hematocrit (%, mean [SD])	35.1 (5.2)	34.8 (5.5)	0.360
Minimum hematocrit (%, mean [SD])	27.2 (5.7)	27.4 (5.6)	0.522
Maximum lactate (mmol/L, median [IQR])	2.3 (1.5, 3.8)	2.9 (1.8, 5.8)	<0.001
Maximum BUN (median [IQR])	21 (15, 32)	30 (20, 46)	<0.001
Maximum creatinine (μmol/L, median [IQR])	1.1 (0.8, 1.6)	1.4 (0.9, 2.4)	<0.001
Maximum INR (mean [SD])	1.6 (1.1)	1.9 (1.8)	0.001
Maximum APTT (median [IQR])	35.2 (29, 48.2)	39.5 (30.6, 71.0)	<0.001
Maximum PT (median [IQR])	15.2 (13.7, 17.3)	16.1 (14.1, 20.0)	<0.001
Maximum glucose (mg/dL, median [IQR])	172 (142, 207)	175 (140, 222)	0.194
Minimum glucose (mg/dL, median [IQR])	97 (80, 117)	101 (78, 121)	0.271

*SD, standard deviation; IQR, interquartile range; BMI, body mass index; SOFA, sequential organ failure assessment; SAPS II, Simplified Acute Physiology Score II; OASIS, Oxford Acute Severity of Illness Score; BP, blood pressure; WBC, white blood cell count; BUN, blood urea nitrogen; INR, international normalized ratio; APTT, activated partial thromboplastin time; PT, prothrombin time*.

### Stepwise Logistic Regression Model

We performed stepwise logistic regression analysis with both forward and backward methods, in which the classifier incorporated 36 variables into the final model. As shown in Table 2, it was found that female (odds ratio (OR), 0.65 [95% confidence interval [CI, 30.6–71.0]), patients with lower BMI (OR, 0.97 [95% CI, 0.95–0.99]), patients with higher PO_2_ (OR for with every 10% increment, 0.98 [95% CI, 0.96–0.99]), and oxygen saturation (SpO_2_) (OR, 0.78 [95% CI, 0.66–0.91]) had higher possibility to survive through discharge. Conversely, neurosurgery (OR, 2.53 [95% CI, 1.57–4.04]), the complication of multiple comorbidities, especially for coagulopathy (OR, 2.36 [95% CI, 1.49–3.68]), greater values of INR (OR, 1.75 [95% CI, 1.15–2.70]), and sodium (OR, 1.08 [95% CI, 1.03–1.13]) were responsible for increased risk of in-hospital death among ICU patients with postoperative sepsis. Furthermore, higher scores in several prognostic scoring systems, including SOFA (OR, 1.08 [95% CI, 1.01–1.16]), SAPS II (OR, 1.04 [95% CI, 1.02–1.06]), and OASIS (OR, 1.05 [95% CI, 1.03–1.08]), were linked to increased in-hospital mortality.

**Table 2:** Variable selection of stepwise logistic regression model.

**Variables**	**OR [95% CI]**	***P-*value**
**Demographic characteristics**		
Gender female	0.65 [0.48, 0.88]	0.006
BMI	0.97 [0.95, 0.99]	0.012
**Elective surgical types**		
Neurosurgery	2.53 [1.57, 4.04]	<0.001
Thoracic surgery	1.91 [0.93, 3.71]	0.065
**Comorbidities**		
Cardiac arrhythmias	1.31 [0.97, 1.78]	0.079
Peripheral vascular diseases	1.61 [1.14, 2.25]	0.006
Coagulopathy	2.36 [1.49, 3.68]	<0.001
Digestive disorders	2.36 [1.66, 3.34]	<0.001
Anemia	0.68 [0.46, 0.98]	0.044
Mechanical ventilation	0.71 [0.46, 1.11]	0.133
**Prognostic scoring system**		
SOFA	1.08 [1.01, 1.16]	0.027
SAPS II	1.04 [1.02, 1.06]	<0.001
OASIS	1.05 [1.03, 1.08]	<0.001
GCS	1.10 [1.04, 1.17]	<0.001
**Vital signs**		
Maximum systolic BP	0.99 [0.99, 1.00]	0.12
Mean diastolic BP	0.94 [0.91, 0.98]	0.001
Mean BP	1.04 [1.00, 1.08]	0.027
Mean respiratory rate	1.03 [1.00, 1.07]	0.076
**Laboratory findings**		
Maximum WBC	0.97 [0.93, 1.00]	0.067
Minimum WBC	1.06 [1.01, 1.11]	0.015
Minimum BUN	1.01 [1.00, 1.02]	0.097
Maximum creatinine	0.63 [0.39, 0.98]	0.052
Minimum creatinine	1.50 [0.90, 2.57]	0.127
Maximum INR	1.16 [0.97, 1.40]	0.085
Minimum INR	1.75 [1.15, 2.70]	0.009
Maximum APTT	1.01 [1.00, 1.01]	0.008
Maximum PT	0.96 [0.92, 1.00]	0.043
Maximum sodium	1.08 [1.03, 1.13]	0.002
Minimum potassium	0.78 [0.58, 1.06]	0.115
Maximum chloride	0.96 [0.92, 0.99]	0.025
Maximum PO_2_ (with every 10% increment)	0.98 [0.96, 0.99]	<0.001
Mean PCO_2_	0.93 [0.90, 0.97]	<0.001
Maximum SpO_2_	0.78 [0.66, 0.91]	0.002
Minimum SpO_2_	1.02 [1.00, 1.03]	0.001
Minimum pH (with every 0.1 increment)	0.51 [0.35, 0.75]	0.001
Minimum BE	1.10 [1.02, 1.18]	0.015

*OR, odds ratio; BMI, body mass index; SOFA, sequential organ failure assessment; SAPS II, Simplified Acute Physiology Score II; OASIS, Oxford Acute Severity of Illness Score; GCS, Glasgow Coma Scale; BP, blood pressure; WBC, white blood cell count; BUN, blood urea nitrogen; INR, international normalized ratio; APTT, activated partial thromboplastin time; PT, prothrombin time; PO_2_, partial pressure of arterial oxygen; PCO_2_, partial pressure of arterial carbon dioxide; SpO_2_, Oxygen saturation; pH, potential hydrogen; BE, base excess*.

### XGBoost Model

**Figure 2:**
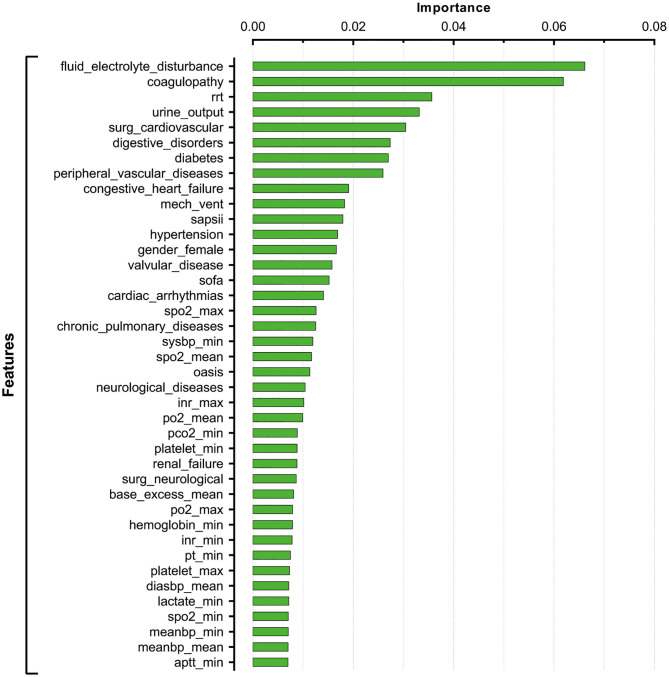
Feature importance derived from XGBoost model.

After tuning and grid search, the hyperparameters applied in the current XGBoost model were as follows: learning rates = 0.01, number of estimators = 1,000, maximum depth of a tree = 5, alpha = 0, and lambda = 0. The importance of feature was assigned by weight which was calculated by the number of times that a feature was used to split the data across all trees. Feature importance revealed the relative contribution of each variable on predicting the in-hospital mortality. As shown in Figure 2, the fluid-electrolyte disturbance and coagulopathy were the top ranked variables that were correlated with in-hospital death among patients with postoperative sepsis, followed by renal replacement therapy (RRT), urine output, cardiovascular surgery, and digestive disorders.

### Evaluation of Model Performance

**Figure 3:**
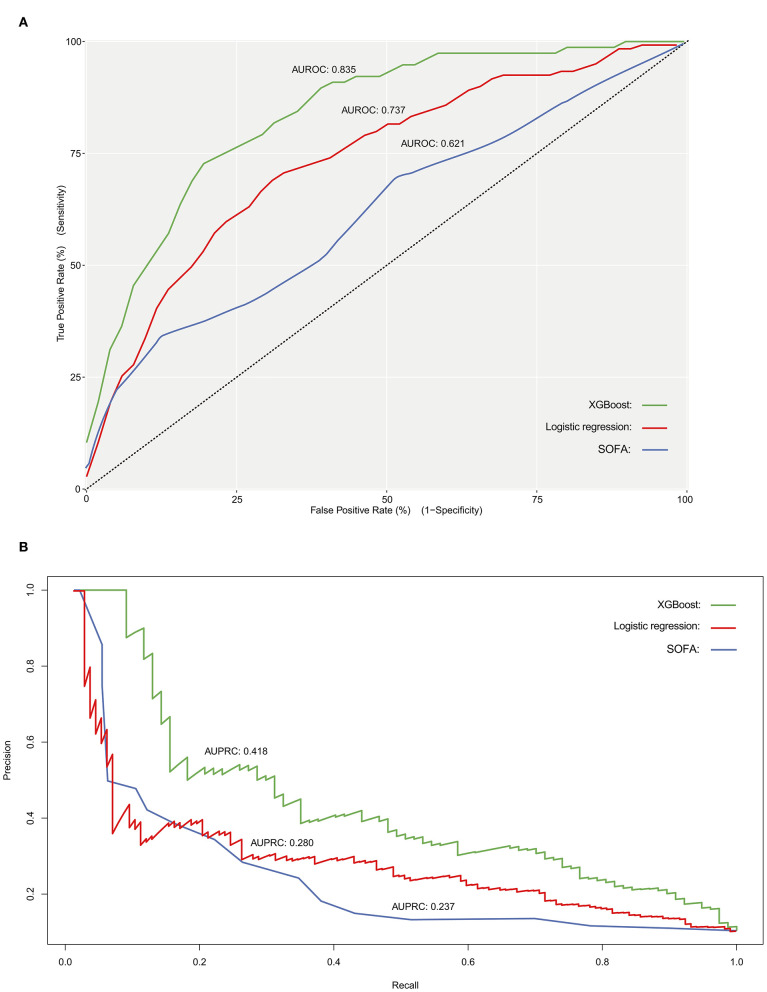
Receiver operating characteristic curve **(A)**, and precision- recall curve **(B)** for evaluating the discriminatory ability of SOFA score (baseline model), stepwise logistic regression model as well as XGBoost model. AUROC, area under the receiver operator characteristic curve; AUPRC, area under the precision-recall curve.

**Figure 4:**
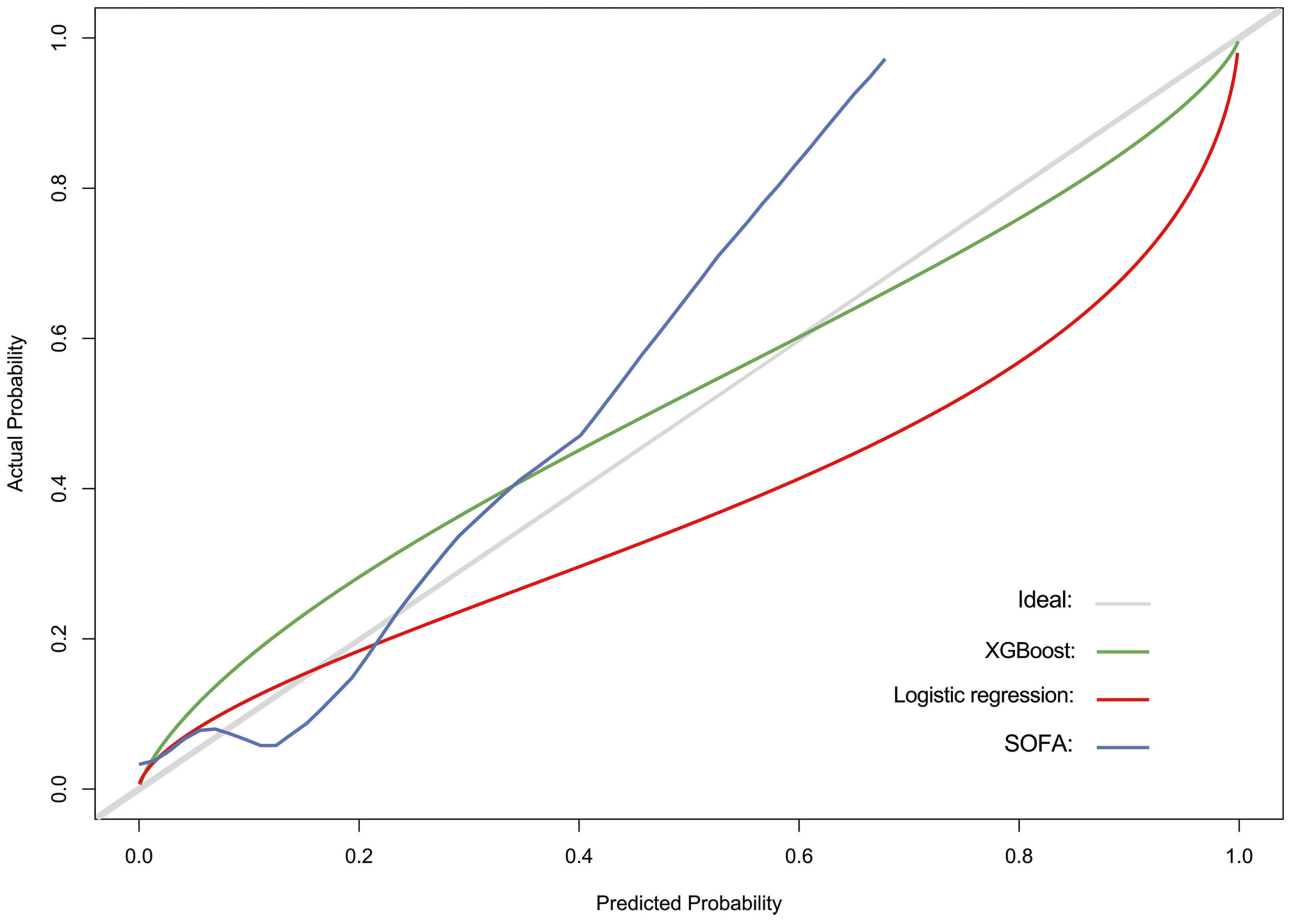
Calibration curve for assessing the goodness of fit for SOFA score (baseline model), stepwise logistic regression model, and XGBoost model.

The discriminatory power of both stepwise logistic and XGBoost models was evaluated by using ROC analysis and c-statistics (calculated by AUROC) in the testing subset. The XGBoost had a significantly higher c-statistics compared to that of the stepwise logistic regression and baseline models (c-statistics, 0.835 vs. 0.737 and 0.621, respectively), suggesting a better discriminative capacity of XGBoost model (Figure 3A). As presented in Figure 3B, the XGBoost model also performed better in terms of precision-recall curve when compared to stepwise logistic regression and baseline models (AUPRC, 0.418 vs. 0.280 and 0.237, respectively). Besides, the accuracy for XGBoost and stepwise logistic models were 0.88 and 0.76, respectively. The recall for XGBoost and stepwise logistic models were 0.10 and 0.75, respectively. Meanwhile, as shown in Figure 4, the calibration curve of models showed that XGBoost presented a greater goodness of fit than logistic regression model and SOFA score.

## Discussion

### Major Findings

In the current study, we identified various clinical indicators that were associated with increased in-hospital mortality among ICU patients with postoperative sepsis. By applying sophisticated machine learning algorithm, we found that fluid-electrolyte disturbance, coagulopathy, RRT, urine output, and cardiovascular surgery were significant features for predicting in-hospital death. In addition, XGBoost model revealed a better performance in discrimination and calibration than that of the conventional stepwise logistic regression model.

### Relation to Other Works

Plenty of evidence have indicated that the development of sepsis is critically involved in short-term and long-term mortality of postsurgical patients (8, 15, 23, 24). A large nationwide epidemiology of patients with elective surgery revealed an increased incidence of postoperative sepsis, ranging from 0.3% in 1997 to 0.9% in 2006, while they found that the in-hospital morality significantly decreased from 1997 to 2006 (44.4–30%) (23). Recently, Ou et al. conducted a population-based analysis in patients who underwent coronary artery bypass grafting (CABG) surgery, and they noticed that the incidence of postoperative sepsis was ~2%, and the mortality of those patients admitted to public hospital and private were 11.9% and 18.3%, respectively (15). In a retrospective analysis by Mørch et al., researchers focused on the clinical outcomes of patients who developed postoperative sepsis after hip fracture surgery. They documented a 30-day mortality of 15.8% among those patients, which was significantly higher than patients without postoperative sepsis (24). In our study, we identified an in-hospital mortality of 10.7% among ICU patients who developed postoperative sepsis. We observed an evident decline in mortality rates among surgical patients with sepsis over the past decades, while the morbidity rates showed sustained increase. The reduction of overall mortality rates might be attributed to the progress in perioperative care and extensive use of antibiotics. Meanwhile, the mortality of patients with postoperative sepsis was disparate from that of the other types of septic patients, which could be explained by different clinical settings and co-morbidities state.

### Clinical Implications

The XGBoost model is capable of accurately predicting in-hospital death among patients with postoperative sepsis. Although several studies have identified the risk factors for the short-term or long-term mortality of septic patients following major operations, few of them establish feasible models to predict clinical outcomes of those patients. Unlike other types of sepsis, postoperative sepsis had some unique characteristics in both etiology and pathophysiology, which made it a specific subset (5). Therefore, it is of great importance to early recognize patients with postoperative sepsis who are at high risk of death and to identify preventable indicators. Since the recent advancements in machine learning techniques, the magnitude of variables and indicators that can be processed is largely enriched. Taken together, advanced machine learning algorithm allows us to establish a more optimal model that performed better in comparison to the conventional generalized linear models. By applying such models, physicians, and care givers could be alerted by the time when ICU patients are complicated with postoperative sepsis, thereby employing efficient yet personalized therapeutic strategies. Although the effectiveness of the XGBoost model had been validated in our study, the model was based on a single center retrospective database. Thus, further prospective cohort studies are required to evaluate the uniformity of this model.

Our results revealed that complication of coagulopathy and coagulation profile at ICU admission, including platelet counts, PT, APTT, and INR, were associated with increased in-hospital death among patients with postoperative sepsis. The occurrence of coagulopathy was commonly seen in septic patients, which was closely related to organ dysfunction and poor outcomes (25, 26). The activation of monocytes and endothelial cells was mainly characterized in the early phase of sepsis and resulted in massive exposure to tissue factors, thereby contributing to the over activation of coagulation and subsequent thrombin generation (27). Concomitantly, anticoagulant pathways, such as protein C system, were impaired by overexpression of proinflammatory cytokines (27). The imbalance between coagulation and anticoagulant pathways can be further augmented by surgical insults, and it leads to the upregulation of plasminogen activator inhibitor and subsequent hyperfibrinolysis (28, 29). Coagulation abnormalities have been reported to induce formation of microvascular clots and disseminated intravascular coagulation (DIC), further resulting in tissue ischemia and organ dysfunction (30, 31). Of note, majority of patients in our study had been exposed with cardiovascular surgery. Some of those patients might frequently receive anticoagulant agents, which could add to coagulation abnormalities. Early implementation of rotational thromboelastometry (ROTEM) and thrombelastography (TEG) appears to be beneficial for patients with postoperative sepsis who are at high risk of death (32, 33). As documented in large randomized controlled trials (RCTs), the administration of either antithrombin III or human recombinant thrombomodulin could improve short-term mortality among septic patients, but no trails specifically targeted patients with postoperative sepsis (34, 35). The results of our study suggested that secondary analyses of previously published RCTs and future large trails were both favorable for better recognition and treatment of septic patients following major operations. In addition, our models identified that fluid-electrolyte disturbance, sodium and chloride levels were associated with the in-hospital mortality, which could be explained by the deteriorative effects of acidosis on fibrin polymerization and clot integrity (28, 36).

From the present observation, we noticed that ICU patients underwent neurosurgery showed the highest in-hospital mortality compared to those with other types of surgery. Meanwhile, it revealed that neurosurgery was a robust predictor of in-hospital death among patients with postoperative sepsis. Neurosurgical procedures might bring about severe complications, including intracerebral hemorrhage, brain edema, and cerebral ischemia, which showed serious impacts on clinical outcomes of neurosurgical patients (37). Furthermore, neurosurgical insults could affect hypothalamic-pituitary-adrenal axis and hormonal generation, resulting in intractable immunosuppression (38). Therefore, well-performed neurocritical care is warranted for neurosurgical patients, especially for those with postoperative sepsis (39).

### Limitations

There are some limitations to our study. Firstly, the current study was a single center retrospective analysis using publicly available database, which restricted us from identifying the causal relationship between variables and endpoints. Thus, prospective cohorts are needed for further validation. Secondly, there were several potential confounding variables that were unable to be assessed due to severe data missing and other reasons. However, some of the excluded variables might have predictive value for clinical outcomes. Thirdly, we employed XGBoost model, a machine learning-based algorithm that was not widely applied in clinical research. Although XGBoost had a significantly higher accuracy in predicting outcomes compared to generalized linear models, overfitting problem was inevitable. Given that, external validation was required to test its utility. Meanwhile, algorithm used other boosting strategy like Adaptive boosting (AdaBoost) were not tested in the current study, which might prevent us from developing more efficient model for predicting our endpoint. Finally, our study merely focused on the in-hospital mortality of patients with postoperative sepsis, while other outcomes, such as long-term mortality and readmission rates, were also important and needed further investigation.

## Conclusions

In summary, these results suggest that some important features are potentially related to the in-hospital mortality among ICU patients with postoperative sepsis. The XGBoost model is capable of processing large amount of variables and further capturing these complicated relationships, which indeed performs better in mortality prediction compared to stepwise logistic model. Further validation of our model in external datasets can prompt us to early recognize patients with postoperative sepsis who are at high risk of death during hospitalization, and to implement timely yet efficient treatments.

## Data Availability Statement

Publicly available datasets were analyzed in this study. This data can be found here: https://mimic.physionet.org.

## Author Contributions

YYa, CR, and ZX conceived the analysis. RY and XJ extracted all data. XJ, YYu, and GWu undertook and refined the inclusion process. RY, CR, and YYu co-wrote the paper. RY, GWa, YZ, and LL undertook the statistical analyses. YL, PZ, and SZ were consulted for clinical issues. All authors contributed to and revised the final manuscript.

## Conflict of Interest

The authors declare that the research was conducted in the absence of any commercial or financial relationships that could be construed as a potential conflict of interest.

## Supplementary Material

Supplemental Figure S1 The process of tuning hyperparameters for XGBoost model.
