# Supplementary figures and images for: A Machine Learning-Based Prediction of Hospital Mortality in Patients With Postoperative Sepsis
Source: Front Med (Lausanne). 2020 Aug 11;7:445. doi: 10.3389/fmed.2020.00445 (PMC7438711; doi:10.3389/fmed.2020.00445)

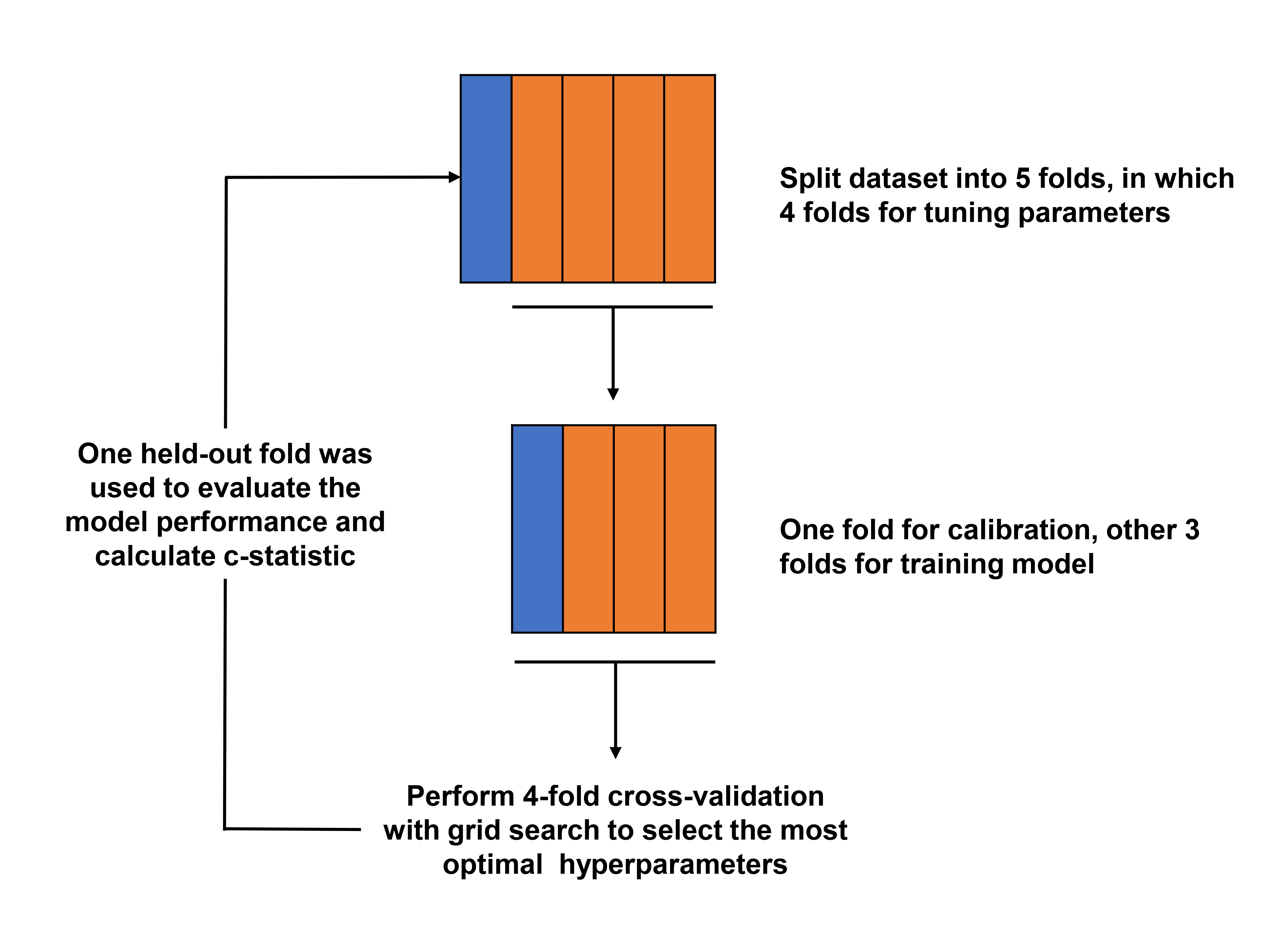

Supplement: Supplemental Figure S1 — The process of tuning hyperparameters for XGBoost model. [file Image_1.TIF]
